# Multi-professional perceptions of clinical research delivery and the Clinical Research Nurse role: a realist review

**DOI:** 10.1177/17449871211068017

**Published:** 2022-04-01

**Authors:** Linda Tinkler, Steven Robertson, Angela Tod

**Affiliations:** Royal College of Nursing Strategic Research Alliance PhD Scholar, Department of Nursing and Midwifery, University of Sheffield, Sheffield, UK; Florence Nightingale Leadership Scholar 2018, NIHR 70@70 Senior Nurse Research Leader; Trust Lead for Nursing, Midwifery and AHP Research, The Newcastle upon Tyne Hospitals NHS Foundation Trust 7315; Programme Director, RCN Research Alliance, Department of Nursing and Midwifery, 7315 University of Sheffield, Sheffield, UK; Professor of Older People and Care, Department of Nursing and Midwifery, University of Sheffield, Sheffield, UK

**Keywords:** clinical research, clinical research nursing, health and social care policy, health services research, inter-professional working, nursing careers, nursing roles, professional identity, realist methods, realist review

## Abstract

**Introduction & Background:**

The delivery of clinical research and the Clinical Research Nurse (CRN) role is fundamental to the wider health agenda, yet both remain misunderstood outwith research teams.

**Methods:**

A realist review was conducted to identify factors that influence how clinical research is perceived by healthcare professionals operating outside NHS clinical research teams. Keyword searches were undertaken across four healthcare databases including grey literature, with iterative snowball searching. Data were extracted from 42/387 sources. Coding generated 3664 extracts of text across 160 themes. Theories generated were presented as ‘If-Then’ statements.

**Results:**

Thirteen theory statements described factors that may influence how clinical research is perceived by healthcare professionals operating outside clinical research teams across three contextual levels:

• Micro: Individual characteristics/behaviours/CRN perceptions
• Meso: Interpersonal relationships at the interface between CRN roles and healthcare delivery
• Macro: Systemwide/infrastructural/cultural issues impacting clinical research delivery.

**Conclusion:**

Factors at micro, meso and macro level contexts may influence how clinical research is perceived by healthcare professionals operating outside clinical research teams. This has the potential to affect the success of clinical research delivery. Meso level theories regarding the perceptions of healthcare professionals outwith research teams may provide insight. Empirical testing of one such theory is underway.

## Introduction

The impact and potential of clinical research in improving prevention, diagnoses, treatment and long-term health related outcomes for the UK population is widely acknowledged across the NHS. The fundamental responsibility of every NHS organisation to engage with research has been clearly stated through various statutory documents and national strategies, the most significant of which led to the formation of the National Institute for Health Research in England in 2006 (DH, 2006; Health Education England, 2011; Health Education England, 2014; DH, 2015; DH, 2017; NHS England, 2019). Research active organisations report lower rates of mortality and research engagement data is now routinely collected during regulatory inspections from the Care Quality Commission (Ozdemir et al., 2015; CQC, 2017).

The Clinical Research Nurse (CRN) role makes an important contribution to realising the aims of the wider health agenda (American Nurses Association, 2016). Despite its continuing evolution and longevity, the CRN role appears to be one of the least understood outwith the field of clinical research delivery in healthcare settings. The lack of understanding presents additional challenges to those in the role, despite numerous attempts to articulate the core elements of the work through various publications (Gordon, 2008; Hardicre, 2013; MacArthur et al., 2014; American Nurses Association, 2016). Persistent complexity, and a perceived continued lack of awareness and visibility in relation to Clinical Research Nursing, have been factors in the variability of role implementation, team structures, support mechanisms, skill mix and leadership across the NHS (Whitehouse and Smith, 2018; Faulkner-Gurstein et al., 2019).

The difficulty for those outside the research arena in understanding the core elements of the CRN role lies partly in the inherent complexity of the terminology associated with research and the infinite variability of the tasks involved in clinical research delivery. Nursing tasks relating to trials are often said to be unique, specific, and additional to routine and familiar nursing skills (American Nurses Association, 2016). Table 1 provides a brief outline of common tasks, however, it does not offer an exhaustive list.

**Table 1.** Common elements of the Clinical Research Nurse role.

*Attracting research to the organisation*
Horizon scanning for opportunities and linking with regional network speciality groups
Supporting the identification and development of new Principal Investigators
Supporting expressions of interest
Leading the coordination of Site Selection Visits
Reviewing and amending schedule of events and other study documentation
*Leading the delivery of research*
Patient eligibility assessment
Patient approach, information sharing, discussion, advocacy, consent and randomisation
Intervention delivery or coordination
Supporting PI with required documentation
Responding to and reporting of Adverse Events
Collection of samples as required
Coordination of couriers
Data collection from baseline through to follow up Accurate recording of source documentation
Site File upkeep
Supporting and coordinating monitoring visits
Ensuring all staff are trained and logged within site file
Administration of study drug/investigational medicinal products
*End of study activities*
Study close down audits
Archiving

The recent COVID-19 global pandemic has exponentially increased public awareness and visibility of the value and importance of research in tackling such health crises (Lancet 2020). Anecdotal reports suggest increased interest in the CRN role across clinical settings, however, the causes of such interest and the sustainability of this remains to be seen.

The realist review described here formed the basis of a study that looks to define and challenge the integrity of theories about the CRN role. The aims and objectives of this review, and the overarching study, are to identify factors that influence how clinical research is perceived by healthcare professionals operating outside clinical research teams within NHS organisations. It further aims to consider how these perceptions, and the resulting behaviours, can subsequently impact on the experiences of CRNs and the organisation’s ability to successfully deliver research.

## Methods

The CRN role can be defined as a complex programme of work, operating at a range of levels and across a range of structures. The complexity of the role and the range of layers involved in its implementation, lends itself to exploration using realist methods. Realist methods seek to generate theory about the causal explanations, often described as mechanisms, behind the range of intended and unintended outcomes observed and reported in relation to interventions or events. This is achieved by going beyond describing merely what is happening, to developing a deeper understanding of why. This is particularly relevant where people and behaviours are involved (Vincent and O’Mahoney 2016, cited in Cassell, Cunliffe and Grandy 2016; Wong et al., 2013).

This realist review utilised the six stages described by Weetman et al. (2017).

### Locating existing theories (Stage 1)

To locate existing theories about the CRN role, initial scoping searches were conducted during January and February 2019, using defined keyword searches (Table 2). This enabled a greater understanding of the breadth of the literature, helped to sift out inappropriate keywords, identified gaps within the literature, and aided in achieving specificity in the formulation of the overarching research question for the study.

**Table 2.** Scoping review literature search strategy template.

Search question	What is known about the understanding of clinical research nurses and its impact on recruitment and retention of patients to studies?
Initial search terms	*Initial search:* ‘Clinical Research Nurs*’ ‘Clinical Trial*’ *‘Clinical Research’* ‘Research Nurs*’ ‘Clinical Trial* Nurs*’ ‘Research Pract*’ *‘Research Delivery’* Research AND Nurs* ‘Patient recruitment’ *‘Patient retention’* *‘Trial retention’* Then search separately for: *‘Professional Identity’* Attitude* Understand* Perception* Awareness *Behaviour* *Development* Experience* Combine each with the initial search terms
Sources to be searched	*Databases* •Cochrane Library •HDAS •PubMED *Grey literature* •British Library ETHOS (e-theses online service) •Health Foundation •NIHR Website •The King’s Fund •Key contacts (using known networks of other researchers undertaking relevant work)
Part of journals to be searched	Title & Abstract Keywords
Years of search	No limits initially Papers will be ordered chronologically, and review will take account of key changes in policy over time e.g. introduction of NIHR
Language	English
Types of studies to be included	•Qualitative •Quantitative •Mixed Methods •Policy/strategy documents and frameworks •Narrative and opinion pieces •Editorials
Inclusion criteria	•Includes reference to nursing as key part of trial/study delivery •Related to delivery of clinical trials/research for others •Papers describing single study teams or broader organisational work •UK based mainly, however, international papers will be included for context and background depending on setting and health system •Primary, Secondary, Tertiary and Industry settings •References recruitment of patients to studies
Exclusion criteria	Nurses conducting own research Non nursing related studies

Theories and associated Context, Mechanism, Outcome configurations were developed from this initial scoping work. In realist methodology, theories are designed to include ‘If-Then’ statements which identify the intended outcome by expressing ‘if we do x then the outcome will be y’. Pawson et al. (2004) articulate that the data to be collected in this stage should not relate to the *‘efficacy of the intervention but to the range of prevailing theories and explanations of how it was supposed to work – and why things “went wrong”’* (p. 16).

Data collected through the initial scoping exercise included information on what the role of the CRN is intended or perceived to be by those in the role, how it has evolved over recent years (in the UK), how the CRN as an intervention is aimed at supporting and positively impacting on the delivery of research in healthcare, and the challenges described by CRNs in practising within their roles.

The scoping exercise uncovered evidence of factors with the potential to impact on the perecpetions, experiences and practice of CRNs. These were attributed to corresponding layers of theory related to individual behaviours (micro), interpersonal relationships and the context in different clinical settings (meso) and institutional, infrastructural, and cultural (macro) level challenges. This contextual framework, serves as a useful heuristic device, through its ability to illustrate varying levels and types of interaction in their contextual setting. This is a common, well recognised and long debated analytical framework in the social sciences (Serpa and Ferreira 2019).

The theories emerging from existing literature appeared to relate to three categories of influencing factors that have the potential to impact on the success of the CRN in undertaking their role and delivering research; social, emotional and physical. Table 3 illustrates these categories in more detail (Spilsbury et al., 2008; Stobbart, 2013; NIHR, 2016; Jones, 2017; Kunhunny and Salmon, 2017; McFadyen and Rankin 2017; Gardner, 2018; Hill, 2018; Tinkler et al., 2018; Tinkler and Robinson 2020).

**Table 3.** Influencing factors with the potential to impact on the CRN Role.

Social influences	Emotional influences	Physical influences
Approaches to communication Concept of etiquette Incentives culture Emotional Labour Gatekeeping behaviours	Perceptions of target based culture Job satisfaction Morale Concept of resilience	Trial complexity Lack of access to or sharing of facilities, rooms and equipment The transient nature of the CRN role and related research studies Physical separation from clinical teams

The evidence located through stage one assisted in clarifying what the CRN role is intended to be, what the indented outcomes are, and what does not seem to work in terms of its implementation in practice. The iterative process of searching and refining theories was continued through to stage 2.

### Search strategy (Stage 2)

The aim of the search at stage 2 was to test the following theory generated by scoping searches at stage 1:

*If* physical, social and or emotional barriers exist in the wider clinical environment, *then* this may impact on the ability of key colleagues to enable, support and promote research in their clinical area. This could negatively impact on the morale and job satisfaction of CRNs and affect working relationships at the interface between the CRN and key colleagues outwith the research team. This could lead to reduced capacity to deliver research and reduce research opportunities offered to patients as part of their clinical pathway.

Utilising the initial scoping searches, informal discussions with experts in the field and with key stakeholders, searches were *‘progressively extended and refocused based on the identified sources’* (Brennan et al., 2014, p. 7). Specific articles identified in reference lists and ‘cited by’ searches enabled the refinement of the initial scoping review to a manageable and specific data set. Two key ‘words’ were found to yield the most relevant literature: ‘Clinical Research Nurs*’ and *‘Research delivery’*.

Whilst the document searching and retrieval process is a key element of the realist approach, other relevant data can be included if identified from sources such as social media, dialogue with experts, TV and radio programmes, online information held in relevant websites and newspaper articles (Emmel et al., 2018). This approach enabled the identification of two further articles held on the NIHR website and a set of videos forming an online resource aimed at sharing the experience of nurses, midwives and allied health professionals (NMAHPs) in research (University of Oxford, Health Experiences Research Group 2019).

### Document selection (Stage 3)

In line with the realist approach, documents and data were selected based on two key factors:

1. Relevance to the aims of the study
2. The potential of added understanding to the existing knowledge base about the intended impact of the CRN role and what does or does not work in its implementation.

Documents and data included empirical studies (qualitative, quantitative and mixed methods), narrative opinion pieces and individual commentaries. A number of policy and strategy documents and frameworks were also collected and reviewed. Whilst these were not deemed appropriate to include in stage 4, they were useful and retained in order to inform the background and foundations for the overarching study. Searching did not identify any relevant social media dialogue, TV reports or newspaper articles. However, discussions with key stakeholders and experts (regional and national NIHR, CRNs and other researchers in this field) did contribute to this stage of the review and directed some of the searching to include specific papers and viewpoints.

### Data extraction (Stage 4)

Data were extracted via an iterative combination of reflective note-taking, highlighting and annotation of sections, and recording document characteristics using Quirkos^©^ software. Papers were categorised by evidence type, main theme of paper, country and setting, and the approach or methodology. Weetman et al. (2017) describe this approach as useful in collecting descriptive information to enable the grouping of documents during review, whilst utilising recognised realist note-taking techniques to achieve data extraction.

During the review, theories were extracted in relation to how the CRN role is intended to work, characteristics required to be successful as a CRN, the views of stakeholders the role is required to interact with, and factors that appear to demonstrate success and failure and why. Extracts of text related to these subject areas were highlighted, noted and broadly labelled.

Throughout data extraction, documents were quality appraised for rigour (Pawson et al., 2004). Realist approaches support the fundamental principle of confirming the quality of data, however, reject the traditional hierarchical approach. It is also important to note that although rigour and relevance were assessed, the exclusion of an entire document based on rigour alone is not advised due to the ability of different sections of different documents to contribute to the evidence base for theory testing and refinement (Pawson et al., 2004).

### Data synthesis (Stage 5)

Data synthesis adopted the approach described by Pawson et al. (2004) which aims to question or confirm theory integrity and search for rival theories.

This approach enabled a focus on specific influencing factors in the implementation of the CRN role in relation to the aims of the study and the theory described in stage 2. Data were synthesised to question the integrity of the current approaches to implementing the role in the NHS; including the social, physical and emotional influences described as emerging from the literature. This enabled the review to establish what it is about the implementation of the CRN role that works (or doesn’t work), for whom, in what circumstances, and why. Data extracted from included sources was used to question and refine the initial theory and identify the potential causal mechanism(s) and the context(s) in which those mechanism(s) might be triggered (Brennan et al., 2014).

### Refine theory (Stage 6)

The final stage of the review involved stakeholder engagement to share and refine the theories derived from the synthesised data in preparation for the empirical stage of the study. Stakeholder perspectives are an important element of refining the final theory(ies). They provide the opportunity to access expert knowledge of the content through a process of checking that the theory(ies) arising from the review match the experiences of CRNs in practice (Weetman et al., 2017).

The opportunity was taken to share early findings and enable questions and clarification from stakeholders at a single point in time, via an interactive conference event attended by a wide range of practising CRNs, their leaders and colleagues. This provided an excellent opportunity to influence interpretation and refinement of the findings in relation to the overarching theory to be taken forward into the empirical elements of this work.

## Findings

**Figure 1.**
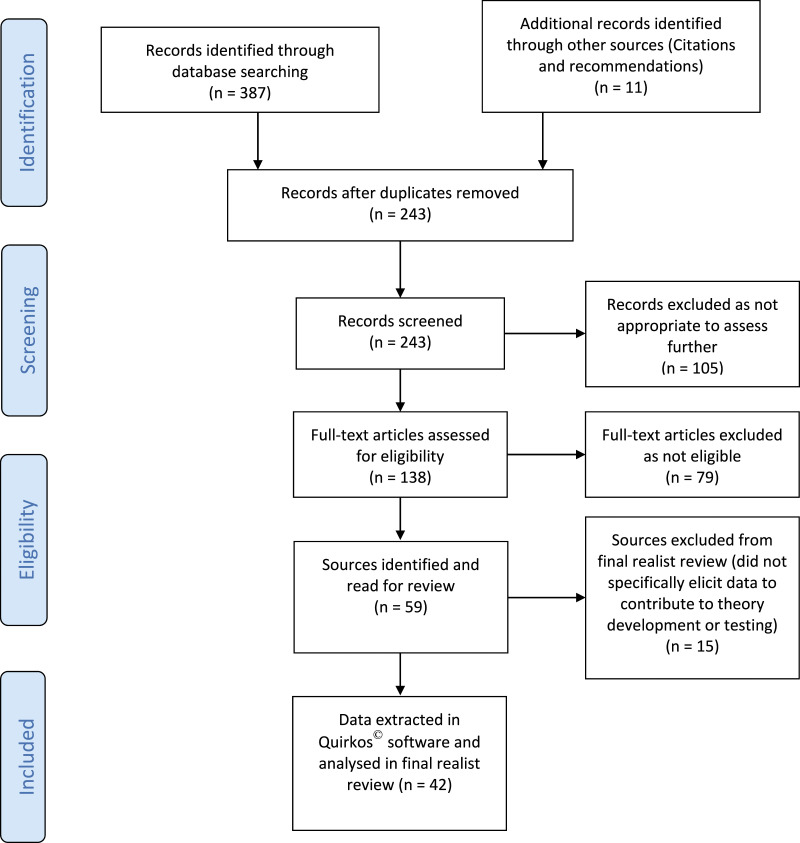
Document flow diagram.

Figure 1 provides a flow diagram to illustrate the final searches undertaken at stage 2 and the selection and exclusion of sources for the review. A Supplementary Table (S1) is available containing document characteristics of the coded papers. A total of 59 papers and data sources were initially located during scoping. During data extraction, all 59 were read, however, 42 were transferred into the Quirkos^©^ software, coded and annotated for the purposes of the realist review. The remaining 15 were retained for interest, future reference, background relevance, or further analysis, as the empirical work progresses. Initial coding generated 3644 extracts of text coded against 160 themes. Themes were further refined and merged, reducing this number to 124 overarching themes. Larger overarching themes were generated in relation to; defining the CRN role, the required skills and competencies, influencing factors in relation to the success of the role, and the research perceptions of the CRN and other staff. A patient related theme was also generated with sub-themes of how patients benefit from research, the nurse patient relationship, and the CRNs’ perceptions of patient motivations to participate in research. Themes were also generated in relation to the overarching research culture and attitudes to research in the NHS, target driven cultures and specific training for the CRN role.

A handful of empirical studies was found relating to CRN perceptions and experiences. These built on existing narrative accounts and provided observed evidence of the experiences described by CRNs. In research carried out by Kunhunny and Salmon (2017), Hill (2018), Tinkler et al. (2018), Tinkler and Robinson (2020) and McCabe et al. (2020), CRNs reported a range of positive experiences in relation to the role. The most significant of these included: being at the forefront of improving and changing practice, care and patient outcomes; increased autonomy; the development of specialist clinical or research related skills; and the increased amount of time they perceived they were able to afford to patients when practising in their role.

This realist review identified the key elements of the CRN role as one of a communicator, boundary spanner, advocate and influencer, in addition to the varied nature of the clinical demands associated with the range of research being delivered. To be successful, the CRN is required to influence across departments and professions whilst advocating on behalf of patients and carers, thereby balancing the preferences of patients with the complexities of implementing discrete research studies across different teams and departments. This has most recently been supported by McNiven et al. (2021) who described an essential part of the role as *‘connecting with and between important groups in research’ *(p. 12).

As the data synthesis progressed, the themes generated were translated into a range of theories with associated Context, Mechanism, Outcome (CMO) configurations. The micro, meso, macro contextual level framework outlined earlier was then used to structure the presentation of these theories.

A total of 13 separate theories with associated CMO configurations were generated during the realist review. These are illustrated in Table 4 and are discussed in the next section.

**Table 4.** Theories and context, mechanism, outcome Configurations.

No	Contextual level	Theory statement (if-then)	Context, mechanism, outcome Configuration
1	Micro	*If the CRN is an effective communicator (influencing, motivational, change management, leadership skills etc.) then he/she may be more successful in engaging key colleagues in supporting or enabling research to take place. This is because positively influencing clinical colleagues can lead to enhanced shared purpose associated with the role of clinical research delivery, subsequently increasing opportunities for patients to be offered research as a core part of their clinical care*	A CRN who is an effective communicator will possess a range of skills to draw on to influence, educate and engage staff in supporting or enabling research to take place, because positively influencing key clinical colleagues through a positive approach, communication style and self-confidence, will lead to a shared understanding and purpose associated with clinical research delivery in the clinical area. This may subsequently increase job satisfaction for the CRN, foster closer working relationships with key colleagues, increase the levels of research related knowledge and confidence in key colleagues and ultimately lead to increased opportunities for patients to be offered research as part of their clinical care
			A CRN who does not possess effective communication skills may inadvertently disengage key staff in relation to the value and utility of supporting clinical research delivery in the clinical area. This may subsequently damage potential for team working, decrease job satisfaction for both the CRN and their key colleagues and lead to decreased opportunities for patients to be offered research as part of their clinical care
			A CRN who is familiar with the clinical area or staff in the area in which she/he is delivering research, may possess key soft intelligence and benefit from established relationships or knowledge of successful approaches to influencing which may lead to a greater shared understanding and purpose associated with clinical research delivery in the clinical area. This could subsequently increase job satisfaction for the CRN, foster closer working relationships with key colleagues, increase the levels of research related knowledge and confidence in key colleagues and ultimately lead to increased opportunities for patients to be offered research as part of their clinical care
2	Micro	*If the CRN has high levels of resilience then he/she may more effectively cope with and or overcome the challenges associated with transitioning into the role, leading to increased productivity and confidence in practice, increasing opportunities for patients to be offered research as part of standard care and delivering more studies to time and target*	A CRN with high levels of resilience may more effectively manage the challenges associated with transitioning into the role, leading to increased productivity and confidence in practice, increasing opportunities for patients to be offered research as part of standard care and successfully delivering more studies to time and target
			A CRN with low levels of resilience may less effectively manage the challenges associated with transitioning into the role, leading to decreased productivity and lower confidence in practice, decreasing opportunities for patients to be offered research as part of standard care and reducing potential to deliver studies to time and target
3	Micro	*If the CRN feels adequately supported in the role then he/she may experience an increased sense of fundamental value associated with their role in delivering research, leading to greater confidence, intrinsic motivation and effort to successfully deliver in the role*	In a context of feeling adequately supported in the role, the CRN may experience an increased sense of value leading to greater intrinsic motivation and effort to successfully deliver in the role
			In a context of feeling adequately supported in the role, the CRN may be more likely to have higher levels of self-efficacy and confidence, enabling more effective communication with key colleagues, fostering a research aware and active culture, ultimately increasing opportunities to introduce research opportunities to patients
4	Micro	*If the CRN does not feel adequately understood or supported in the role then he/she may experience a reduced sense of fundamental value associated with their role in delivering research, leading to increased isolation, lower confidence levels, reduced intrinsic motivation and reduced job satisfaction. This could impact on subsequent potential to successfully deliver in the role*	In a context of feeling inadequately supported in the role, the CRN may experience a reduced sense of value leading to lower levels of intrinsic motivation and effort to successfully deliver in the role
			In a context of feeling inadequately supported in the role, the CRN may develop lower levels of self-efficacy and confidence, hindering the ability to effectively communicate with key colleagues. This may impact on the ability to foster a research aware and active culture and may ultimately decrease opportunities to introduce research opportunities to patients and deliver studies to time and target
5	Meso	*If key colleagues do not understand the importance, value and utility of research to their role, their patients, or the wider NHS agenda then they may inadvertently display avoidance of or resistance to research being delivered in their clinical area, thereby hindering positive working relationships with CRNs and reducing access to research opportunities for patients*	In the context where key colleagues are not aware of the importance, value and utility of research to their role, the patients in their care, or the wider NHS, then they may be unaware of the impact of their behaviours on the CRN and the wider research agenda, leading CRNs to feel undervalued, unwelcome and invisible, thereby hindering positive working relationships with CRNs and reducing access to research opportunities for patients
			In the context where key colleagues do not understand the importance, value and utility of research to either their role or the patients in their care, then they may display active resistance to research being delivered in their clinical area, this leads CRNs to feel undervalued, unwelcome and invisible, thereby hindering positive working relationships with CRNs and reducing access to research opportunities for patients
			In the context where key colleagues do not understand the importance, value and utility of research to either their role or the patients in their care, then they may display avoidance in relation to research being delivered in their clinical area, this leads CRNs to feel undervalued, unwelcome and invisible, thereby hindering positive working relationships with CRNs and reducing access to research opportunities for patients
6	Meso	*If key colleagues do not understand the importance, value and utility of research to their role or their patients then they may knowingly display avoidance of or resistance to research being delivered in their clinical area, thereby hindering positive working relationships with CRNs and reducing access to research opportunities for patients*	In the context where key colleagues may not understand the importance, value and utility of research to either their role or the patients in their care, then they may knowingly display avoidance of or active resistance to research being delivered in their clinical area, this will lead CRNs to feel undervalued, unwelcome and invisible, thereby hindering positive working relationships with CRNs and reducing access to research opportunities for patients
7	Meso	*If key colleagues are not interested in (intrinsically motivated by) research then they may display gatekeeping behaviours of avoidance of or active resistance to research being delivered in their clinical area, thereby hindering positive working relationships with CRNs and reducing access to research opportunities for patients, impacting on organisational performance in relation to research activity and culture*	If key colleagues are not interested in (intrinsically motivated by) research then they may display avoidance of or active resistance to research being delivered in their clinical area, this will lead CRNs to feel undervalued and unwelcome thereby damaging positive working relationships and reducing access to research opportunities for patients, impacting on organisational performance in relation to research activity and culture
			In the context of a lack of interest in research from key colleagues, the CRN may deploy tactics to incentivise colleagues to support them in gaining access to space, facilities or patients in the clinical area, the need to deploy such tactics may lead CRNs to feel undervalued and unwelcome thereby damaging positive working relationships and reducing access to research opportunities for patients, impacting on organisational performance in relation to research activity and culture
8	Meso	*If key colleagues are fearful of increased workload as a result of research activity in their clinical area, then they may avoid communicating with the CRN or actively prevent access to facilities or patients, thereby reducing opportunities to introduce research as part of standard care and affecting the CRNs ability to deliver a study to time and target*	If key colleagues are fearful of increased workload as a result of research activity in their clinical area, then they may, avoid communicating with the CRN this may lead CRNs to feel burdensome, unwelcome, ostracised and undervalued, reducing opportunities for shared learning in relation to research delivery within clinical practice and also affecting the ability to introduce research to patients as part of standard care
9	Meso	*If key colleagues are fearful of risk to their patients as a result of research activity in their clinical area, then they may avoid communicating with the CRN or actively prevent access to facilities or patients, thereby reducing opportunities to introduce research as part of standard care and affecting the CRNs ability to deliver a study to time and target*	If key colleagues are fearful of risk as a result of research activity in their clinical area, then they may, *avoid communicating with the CRN* this may lead CRNs to feel burdensome, unwelcome, ostracised and undervalued, reducing opportunities for shared learning in relation to research delivery within clinical practice and also affecting the ability to introduce research to patients as part of standard care
			If key colleagues are fearful of risk as a result of research activity in their clinical area, then they may *actively prevent CRNs from accessing facilities or patients* this may lead CRNs to feel burdensome, unwelcome, ostracised and undervalued reducing access to the ability to introduce research to patients as part of standard care
10	Meso	*If Clinical Research is (seen as) a priority for senior management then support for the CRN including development and progression opportunities in relation to the role will be integral to the trust’s ongoing research strategy, leading to better working relationships with key colleagues and increased research capacity*	In the context where Clinical Research is (seen as) a priority for senior management then support for the CRN including highlighting the importance of the role, development and progression opportunities will be integral to the trust’s ongoing research strategy. This will then lead to increased visibility and awareness of the role in key colleagues enabling greater capacity to deliver research and offer more opportunities to patients to participate in research
11	Meso	*If the CRN role is clinically embedded or is co-located with the clinical team then shared purpose and joined up working is more likely, enabling higher levels of research capacity within the team, greater understanding of the role of the CRN and a culture of offering research opportunities to patients where possible*	If the CRN role is clinically embedded or is co-located with the clinical team then joined up working is more likely, this then leads to a greater understanding of the role of the CRN enabling higher levels of research capacity within the team and fostering a culture of offering research opportunities to patients where possible
12	Macro	*If the National focus (message) is on achieving targets and subsequently attracting funding then trust between individual, organisational and regional/national levels may be eroded and morale may be affected because CRNs feel the focus on patients is lost, leading to lower levels of intrinsic motivation which may impact on productivity in relation to delivering research*	In the context where the national strategic focus is on achieving targets and resulting funding then trust is eroded, and morale is affected because CRNs feel the focus on patients is lost leading to lower levels of intrinsic motivation which may impact on productivity in relation to delivering research
			In the context where the national strategic focus is on achieving targets and resulting funding then CRNs identify with the negative elements of their perceptions of the role of the salesperson, leading to lower levels of intrinsic motivation altered professional identity and reduced morale which may impact on productivity in relation to delivering research
13	Macro	*If the National strategic focus is on promoting the value and potential of research to patients as an integral element of the care pathway* ***,*** *CRNs will feel the focus on patients is strong, leading to higher levels of intrinsic motivation which may impact on productivity in relation to delivering research*	In the context where the national strategic focus is on promoting the value of research to patients, then trust and morale are increased because CRNs feel the focus on patients is strong, leading to higher levels of intrinsic motivation which may impact on productivity in relation to delivering research
			In the context where the national strategic focus is on promoting the value of research to patients, then CRNs identify with the positive elements of the role of advocate and the nurse patient relationship, leading to higher levels of intrinsic motivation and improved morale which may impact on productivity in relation to delivering research

## Discussion

The research related perceptions of healthcare professionals practising outside research roles identified in the literature, mainly related to the concept of nurse-led research and the demands of fitting research into one’s own clinical practice. Little was identified in relation to specific views of research delivery, or the CRN role outwith the views of research teams and CRNs. This demonstrates a clear research gap and suggests the urgent need to explore these currently absent views to enable a deeper understanding of the experiences described by CRNs.

The four micro level theories generated by this review help to confirm previous reports that a combination of intrinsic communication skills, resilience, and perceptions of feeling supported and valued within the role, may directly affect the success of the individual within the role (Tinkler et al., 2018; Tinkler and Robinson 2020). Whilst these factors have been reported in the literature elsewhere, this review has enabled them to be situated within a framework of context, mechanism and outcome, enabling further consideration to be given to their importance in relation to the delivery of research in the NHS. Whilst resilience is the subject of ongoing debate in nursing, evidence emerging from the empirical data reviewed here suggests that those CRNs possessing higher levels of resilience may more effectively cope with, and/or overcome the challenges associated with transitioning into the role (Traynor 2018; Tinkler et al., 2018).

The seven meso level theories highlight a multifaceted range of potential mediators of success, ultimately generated by human agency. This term was defined by Bandura (1989, 2001) as the capability of humans to be able to influence one’s own functioning and the courses of events through one’s own actions.

When the concept of human agency is considered specifically in relation to the CRN role and its implementation, the individual beliefs, past experiences, environment, organisational culture and decisions made by actors within and outside the role are played out through the visible behaviours displayed at the interface between research delivery and clinical practice/care provision. These challenges can be better understood when viewed through the lens of social structures. As such, interactions between, and impacting on, the CRN and colleagues outside of the research team can range from choosing to embrace and promote research, to avoiding, displaying scepticism and exhibiting gatekeeping behaviours in relation to the role. These directly impact on the CRN and affect their subsequent behaviours in relation to such interactions.

The recent work of McNiven et al. (2021) supports these findings, building a strong link between the articulated professional identity of Research Nurses, Midwives and AHPs (R-NMAHPs) and the wider contexts in which they work. This includes groups of individuals such as non research active clinical colleagues. McNiven et al. (2021) describe a sense of alienation and rejection from clinical colleagues, which affects their ability to absorb boundary spanning activities, despite the positive potential of this key element of the role.

According to Pawson and Tilley (1997), the successful implementation of any ‘*programme*’ relies on the very minimal requirements of cooperation and non-disruption. The perceived relevance of a programme, intervention, policy or strategy to an individual is also an important marker of whether they will be drawn to, act in support of, or experience change as a result of its implementation. Using Pawson and Tilley’s theoretical proposition helps to explicitly link the notion that individuals outwith research teams need to, as a minimum, demonstrate cooperation with research teams to enable the successful delivery of research. That is to say, where cooperation as a bare minimum does not exist, the chances of success are reduced. A key inference from this review is therefore that interactions at the interface between research delivery and care delivery, and leadership and organisational culture, may act as mediators in the potential success of research delivery in the NHS.

Leadership and culture are terms used regularly in discussion and debate related to the NHS, the quality of patient care and its performance in relation to government targets. A range of evidence describes the impact of leadership and culture on the cohesiveness of teams, the behaviour of NHS staff and the resulting quality of care (Turnbull James, 2011; West, 2012; Francis, 2013). In previous work, Tinkler et al. (2018), and Tinkler and Robinson (2020), identified the impact of culture and leadership specifically in relation to the research agenda. They suggest that visibility, awareness, value and organisational context have the potential to impact on both staff within and outside research delivery teams, ultimately mediating attitudes towards research and the resulting ‘research culture’. The meso level theories generated by this review provide insight into the key interactions required for the success of research delivery.

At a macro level, in the context of continued nursing shortages and wider workforce challenges in the NHS, maintaining adequate research delivery capacity presents a significant challenge, inevitably affecting the recruitment and retention of CRNs (Faulkner-Gurstein et al., 2019). The NHS People Plan identifies the nursing shortage as both the most significant and most urgent of the challenges faced, and importantly outlines the key part the nursing role plays within the *‘multiprofessional team needed to deliver the NHS Long Term Plan...’* (NHS England, 2020, p. 20). The plan acknowledges the need for a ‘*multifaceted and carefully coordinated strategy’* (p. 20) to include, amongst other key ingredients, improved retention of nurses and the clear provision of equitable career development opportunities to meet the needs of both the workforce and the changing requirements of our patient populations. Research and effective research delivery remain integral to these aims, though are not forefronted within the plan.

Weaved throughout the NHS People Plan is a fundamental thread; a commitment to making the NHS the best place to work through inclusive and compassionate leadership. The plan identifies the importance of the right type of behaviours expected in our interactions with each other, and across the system. A link can be made here between the NHS People Plan’s commitment to inclusive and compassionate leadership, and the potential influence of culture, leadership and the resulting behaviours displayed during interactions between CRNs and colleagues outside of the research team.

Adding to the omnipresent workforce issues is continued evidence that few trials are able to recruit and retain the required number of participants within the planned timeframe in order to address the primary outcome measure and answer the original research question (Campbell et al., 2007; Donovan et al., 2014; Gardner, 2018; Treweek et al., 2018; NIHR 2020). Increasingly, ‘research on research’ is being undertaken to explore what is termed *‘recruitment to time and target’* and the efficiency of clinical trials in answering research questions (Preston et al., 2016; Houghton et al., 2017; Gardner, 2018). The focus of this emergent evidence base is mainly related to the practicalities of trial design, methodology, and approaches to training site investigators (Donovan et al., 2014; Mills et al., 2018; Gardner, 2018; Rooshenas et al., 2018, 2019). Within this field there remains relatively limited acknowledgement of the complexity of human agency and the wider context-related complexities of implementing a study protocol which is designed at one site (in a specific context) but then delivered at other sites through different teams with different challenges.

Such macro level challenges impact on the individual CRNs at micro levels in relation to morale, job satisfaction and intention to remain in post. They also have the potential to influence the organisational capacity to offer research opportunities to patients and the resulting success of research projects delivered within the clinical area. This poses a significant risk in relation to the wider government ambition to ensure the UK has a flourishing Life Sciences industry and that the UK is seen as a preferred location in which to undertake clinical research. There are also further reputational consequences in relation to research and clinical care related league tables and their associated quality ratings (DH, 2017; NIHR, 2016; NIHR 2020).

In an online blog written for the BMJ, Leary (2019) stated that *‘healthcare is a human activity delivered by humans... and trying to model skills, task delivery, or any other abstraction of the work is unlikely to meet with success’.* Leary suggested there is a necessity to explore workforce needs in relation to the populations they serve, with workforce design driven by that demand. Leary also stated that courage is required to enable emancipation from ‘activity’ being the primary measure of success in health and suggesting that outcomes for patients and workers should also be considered when thinking about workforce planning.

## Strengths and Limitations

The aim of this review was to draw out the underlying mechanisms that may shed light on the experiences described by CRNs, the perceptions they articulate about practising within their roles, and their interactions with other professionals as they go about their work. The majority of literature relating to perceptions of the CRN role, and research delivery in the NHS, is understandably presented by CRNs, sharing their passion for the role, aiming to create debate, raise awareness and visibility and attracting others into the role by highlighting the main elements of the work. The lack of literature regarding views of health professionals outside of the role is not surprising, however, it must be acknowledged that the perspectives of these individuals is currently absent and a more balanced view of the context in which research is delivered in today’s NHS should be sought.

The majority of literature generated by CRNs thus far has historically been subjective in nature, or generally specific to single research studies or discrete clinical specialities. Case studies, narrative discussion pieces and reflective accounts form the majority of the evidence base accessed for this review (Gordon, 2008; Gibbs and Lowton, 2012; Hardicre, 2013; NIHR 2016). Whilst this could be viewed as a limitation, in realist approaches the lived experience and therefore expertise of those embedded in a particular context has the potential to uncover key clues as to why something works or does not work, for whom and in what circumstances. Such evidence may not have been acceptable to include in a standard systematic review approach.

The approach to data extraction in this review included conventional methods of extracting, categorising and annotating excerpts of text from the sources selected, to enable the process of theory extraction. The methodology and transparency of realist reviews has been reported to demonstrate low levels of uniformity and transparency, with recommendations that further specific methodological guidance would be beneficial to promote this (Berg and Nanavati 2016). The realist review reported here aimed to promote transparency by following the RAMESES publication standards for realist review, however, as the methodology and literature related to realist reviews continues to evolve, it is important to note that this realist review could have been improved. For example, Cooke at al (2018) conducted a realist review, generating indicative ‘If-Then’ statements for each paper analysed. This approach may have been useful to adopt in the current review and could be considered in the future.

## Conclusion

This novel realist review has served as a useful opportunity to utilise realist methodology in this subject area and to draw together a variety of literature, both empirical and non-empirical, in relation to the CRN role. The nature and multiplicity of the challenges outlined provides an indepth insight into the complex reality of implementing the delivery of research in the NHS, identifying what works and what is perceived to not work in the eyes of the CRN, and the contexts in which such mechanisms are thought to be triggered.

## Implications for practice and recommendations for future research

The range of challenges with the potential to impact on the success of research delivery in the NHS remains wide and varied. The review conducted here suggests extensive work is required to optimise research delivery and to reduce the manifestation and complexity of the challenges described as impacting on successful research delivery. A key contribution to optimising research delivery potentially lies in the decisions made by actors both within and outside the research arena. These are often played out through the visible behaviours displayed at the interface between research delivery and clinical practice and care provision. This review has identified a gap in the literature in relation to the perceptions of stakeholders external to the research team. By exploring these views, the theories generated can be tested and either confirmed or refuted, enabling clear recommendtions to be made in relation to the future of research delivery roles in the NHS.

**Key points for policy, practice and/or research**

• Realist methodology provides a useful framework to explore the implementation of research in the NHS because it accounts for contextual factors that other methods may not include.
• The perceptions of stakeholders external to the research team should be investigated to enable their views to be heard and understood.
• The thirteen micro, meso and macro level theories generated require further exploration in relation to how research is implemented in the NHS.
• The context of research delivery may have changed in light of the COVID-19 pandemic; this should be further explored to ensure the resulting opportunities are maximised.

## Supplemental Material

sj-pdf-1-jrn-10.1177_17449871211068017 – Supplemental Material for Multi-professional perceptions of clinical research delivery and the Clinical Research Nurse role: a realist review Click here for additional data file. Supplemental Material, sj-pdf-1-jrn-10.1177_17449871211068017 for Multi-professional perceptions of clinical research delivery and the Clinical Research Nurse role: a realist review by Linda Tinkler, Steven Robertson and Angela Tod in Journal of Research in Nursing
