# Supplementary material for: Multi-professional perceptions of clinical research delivery and the Clinical Research Nurse role: a realist review
Source: J Res Nurs. 2022 Apr 1;27(1-2):9–29. doi: 10.1177/17449871211068017 (PMC8980584; doi:10.1177/17449871211068017)
Supplement: sj-pdf-1-jrn-10.1177_17449871211068017 – Supplemental Material for Multi-professional perceptions of clinical research delivery and the Clinical Research Nurse role: a realist review [file sj-pdf-1-jrn-10.1177_17449871211068017.pdf]

Supplementary Table S1 Document Characteristics of papers included in the realist review (January and February 2019)

No	Author, Year (Reference)	Country	Evidence Type	Approach or Methodology	Main Theme of Paper	Setting
1	Boulton MG, Beer S. (2018) Factors affecting recruitment and retention of nurses who deliver clinical research: A qualitative study. *Nursing Open* 5 555–566.	UK	Empirical Study	Focus Groups	Factors influencing intention to remain in post	Multiple Settings
2	Brady O (2017) Clinical research offers a rewarding career option for nurses. *Nursing Times* [online]; 113: 10, 34-36.	UK	Discussion/Opinion Piece	Not Applicable	Influencing perceptions of others/highlighting benefits of the CRN role	Not Applicable
3	Brinkman-Denney, S. (2013) An international comparison of the clinical trials nurse role. *Nursing Management (UK)*, 20(8), 32-40.	International	Literature Review	Systematic Review	Role of the CRN	Multiple settings
4	Brown, J., Barr, O., Lindsay, M., Ennis, E. & O'Neill, S. (2018) Facilitation of child health research in hospital settings: The views of nurses. *Journal of Clinical Nursing*, 27(5-6), 1004.	UK	Empirical Study	Questionnaire or Survey Design	Value, Visibility, Understanding and Awareness of CRN Role	Secondary Care
5	Campbell, T. (1998) Patient-focused care: primary responsibilities of research nurses *British Journal of Nursing* 7 (22)	UK	Narrative Review	Not Applicable	Role of the CRN	Primary Care
6	Coulson C, Grange A (2012) Developing clinical research nurses. *Nursing Times;*	UK	Empirical Study	Mixed Methods	Education, Training &	Secondary Care

	108 (22/23) 23-25.				Development in relation to CRN role	
7	Dunleavy, L., Griggs, A., Wiley, G. and Hughes, M. (2011) Overcoming the hurdles: setting up clinical trials in three UK hospices**.** *International Journal of Palliative Nursing* 17 (3)	UK	Discussion/Opinion Piece	Not Applicable	CRN Perceptions and Experiences	Third Sector
8	Fuchs, B. (2017) Can we talk about power? The King's Fund	UK	Discussion/Opinion Piece	Not Applicable	Leadership and Culture in the NHS	Not Applicable
9	Gelling, L. (2010) Clinical Research Nursing has a bright Future *Nurse Researcher* 17 (2) 3	UK	Editorial	Not Applicable	Influencing perceptions of others/highlighting benefits of the CRN role	Not Applicable
10	Gibbs, C. L. & Lowton, K. (2012) The role of the clinical research nurse. *Nursing Standard*, 26(27), 37-40.	UK	Discussion/Opinion Piece	Not Applicable	Role of the CRN	Secondary Care
11	Goosen, S. (2015) The importance of teamwork in nursing *Professional Nursing Today* 2015;19(3):4-6	UK	Discussion/Opinion Piece	Not Applicable	Importance of teamwork within. Nursing	Not Applicable
12	Gordon, C. (2008) Exploring the new specialty of clinical research nursing. *Nursing Times*, 104(29), 34-35.	UK	Narrative Review	Not Applicable	Role of the CRN	Secondary Care
13	Green L. (2011) Explaining the role of the nurse in clinical trials. *Nursing*	UK	Discussion/Opinion Piece	Not Applicable	Role of the CRN	Secondary Care

	Standard, 25 (22) 35-9.					
14	Hamer S (2015) The nurse's changing role in clinical research. *Nursing Times;* 111: 39, 12-14.	UK	Discussion/Opinion Piece	Not Applicable	Research in Nursing	Not Applicable
15	Hardicre, J. (2013) An exploration of the role of the research nurse and its impact. *British Journal of Nursing*, 22(3), 168-169.	UK	Discussion/Opinion Piece	Not Applicable	Role of the CRN	Not Applicable
16	Hardicre, J. (2013) Developing research nurses: a structured taxonomic model. *British Journal of Nursing*, 22(7), 416-418.	UK	Empirical Study	Scoping Exercise	Education, Training & Development in relation to CRN role	Secondary Care
17	Hemingway B, Storey C (2013) Role of the clinical research nurse in tissue viability. *Nursing Standard.* 27, 24, 62-68	UK	Discussion/Opinion Piece	Not Applicable	CRN Perceptions and Experiences	Secondary Care
18	Hill, G. (2018) *Exploring Clinical Research Nurse's Experiences of working with Clinical Nurses*. Professional Doctorate Queen Margaret University.	UK	Empirical Study	Semi-structured Interviews	CRN Perceptions and Experiences	Secondary Care
19	Houlston, C. (2012) The role of a research nurse in translating evidence into practice. *Nursing Management (through 2013)*, 19(1), 25-8	UK	Discussion/Opinion Piece	Not Applicable	CRN Perceptions and Experiences	Secondary Care

20	Hyland, D. & Moloney, M. C. (2016) Spotlight on clinical research nursing. *World of Irish Nursing & Midwifery*, 24(3), 52-53.	UK	Discussion/Opinion Piece	Not Applicable	Role of the CRN	Not Applicable
21	Jones, H. (2017) *Exploring the experience of Clinical Research Nurses working within acute NHS trusts and determining the most effective way to structure the workforce: A mixed methods study* Doctorate in Nursing (Healthcare) Kings College London	UK	Empirical Study	Mixed Methods	CRN Team Structures	Secondary Care
22	Kunhunny, S. & Salmon, D. (2017) The evolving professional identity of the clinical research nurse: A qualitative exploration. *Journal of Clinical Nursing*, 26(23-24), 5121.	UK	Empirical Study	Focus Groups	CRN Perceptions and Experiences	Secondary Care
23	Larkin, M. E., Beardslee, B., Cagliero, E., Griffith, C. A., Milaszewski, K., Mugford, M. T., Myerson, J. M. Ni, W. Perry, D. J., Winkler, S. and Witte, E.R. (2019) Ethical challenges experienced by clinical research nurses: A qualitative study *Nursing Ethics* 26(1) 172–184	US	Empirical Study	Semi-structured Interviews	CRN Perceptions and Experiences	Secondary Care
24	Lawan, M. (2017) Trials and beyond: role of the cardiovascular research nurse British Journal of Cardiac Nursing 12 (4)	UK	Discussion/Opinion Piece	Not Applicable	Role of the CRN	Secondary Care

25	Ledger, T. (2008) Developing Clinical Research Nurses. *Nursing Management* 15 (2) 28-33	UK	Case Study	Scoping Exercise	Role of the CRN	Secondary Care
26	MacArthur, J., Hill, G. & Callister, D. (2014) Professional issues associated with the clinical research nurse role. *Nursing Standard*, 29(14), 37-43.	UK	Empirical Study	Questionnaire or Survey Design	Education, Training & Development in relation to CRN role	Multiple Settings
27	McCormack, B. (2004) Clinical Research Nurses should be involved in Nursing Research strategies of the future. *Nursing Times* 9 (1) 28-29	UK	Discussion/Opinion Piece	Not Applicable	Influencing perceptions of others/highlighting benefits of the CRN role	Secondary Care
28	McDermott S, Hathaway, K., Saunders, C. (2014) Developing good practice for clinical research nurses. *Nursing Standard.* 28, 26, 40-44.	UK	Narrative Review	Not Applicable	Role of the CRN	Secondary Care
29	McFadyen, J. & Rankin, J. (2017) The Role of Gatekeepers in Research: Learning from Reflexivity and Reflection. *GSTF Journal of Nursing and Health Care.* 4 (1)	UK	Discussion/Opinion Piece	Retrospective reflection	Gatekeeping	Not Applicable
30	National Institute for Health Research. (2016) The Role of the Clinical Research Nurse: In their own words. https://www.nihr.ac.uk/documents/the-role-of-the-clinical-research-nurse/11505	UK	Web page or Blog	Not Applicable	Role of the CRN	Multiple Settings

31	National Institute of Health Research (2019) Keep Making a Difference: Nurses and Midwives https://www.nihr.ac.uk/documents/keep-making-a-difference-nurses-and-midwives/22555#Keep_Making_a_Difference	UK	Web page or Blog	Not Applicable	Role of the CRN	Multiple Settings
32	Norton, C. (2015) Research nurses have a crucial role in delivering patient care. *Nursing Times* Vol 111(19)	UK	Discussion/Opinion Piece	Not Applicable	Role of the CRN	Secondary Care
33	Routlegde, J., Burns, M., Davidson, S. E., Johnson, K., Swindell, R., Khoo, V. (2003) The emerging role of the Cancer Research Nurse in promoting Evidence Based Care in Radiotherapy *European Journal of Cancer 1* (5) supplement 1139	UK	Conference Abstract (Oral)	Prospective and retrospective data review	Importance of Role of the CRN	Secondary Care
34	Smith S, Gullick J, Ballard J, Perry L. A proposed clinical research support career pathway for non-investigators. International Journal of Nursing Practice. 24 (e12641).	Australia	Discussion/Opinion Piece	Not Applicable	CRN Perceptions and Experiences	Tertiary
35	Spilsbury, K., Petherick, E. & Cullum, N. (2008) The role and potential contribution of clinical research nurses to clinical trials. *Journal of Clinical*	UK	Empirical Study	Focus Groups	CRN Perceptions and Experiences	Secondary Care

	Nursing, 17(4), 549-557.					
36	Stephens-Lloyd, A. (2004) The extended role of the clinical research nurse: Building an evidence base for practice. *Nursing Times* 9 (1) 18-27	UK	Discussion/Opinion Piece	Not Applicable	Role of the CRN	Secondary Care
37	Stephenson, J. (2017) Exclusive: New strategy will emphasise role played by research nurses *Nursing Times.* *https://www-nursingtimes-net.sheffield.idm.oclc.org/news/research-and-innovation/new-strategy-will-emphasise-role-played-by-research-nurses-07-09-2017/*	UK	Good News Story/Article	Not Applicable	Value, Visibility, Understanding and Awareness of CRN Role	Not Applicable
38	Thompson Hones, H., Palmer, G. and Whelan, S. (unable to identify year) Day in the life of a research nurse. located via http://www.wales.nhs.uk/sitesplus/documents/863/day%20in%20the%20life%20of%20a%20research%20nurse.pdf	UK	Discussion/Opinion Piece	Not Applicable	CRN Perceptions and Experiences	Secondary Care
39	Tinkler, L., Smith, V., Yiannakou, Y. & Robinson, L. (2018) Professional identity and the Clinical Research Nurse: A qualitative study exploring issues having an impact on participant recruitment in research. *Journal of Advanced Nursing*, 74(2), 318.	UK	Empirical Study	Focus Groups	CRN Perceptions and Experiences	Secondary Care

40	University of Oxford Health Experiences Research Group (2019) *Nurses, midwives & allied health professionals in research-research nurses and midwives experiences of working with clinical colleagues.* https://www.healthtalk.org/experiences-nurses-midwives-allied-health-professionals-research/research-nurses-midwives-and-ahps-experiences-of-working-with-clinical-colleagues	UK	Empirical Study	Semi-structured Interviews	CRN/Research active NMAHPs Perceptions and Experiences of working with clinical colleagues	Secondary Care
41	Whitehouse, C., Smith, H. A. (2018) The Whitehouse Report: Review of research nursing and midwifery structures, strategies and sharing of learning across the UK and Ireland in 2017. The Florence Nightingale Foundation	UK	Service Evaluation	Semi-structured Interviews	Review of Research Nursing and Midwifery CRN Team Structures, the role of the CRN, perceptions, experiences of CRNs and views of those outwith research roles	Multiple settings
42	Wytrykowski S (2019) Evaluation of a primary care clinical research nursing service. *Nursing Times* [online]; 115: 1, 30-32.	UK	Service Evaluation	Questionnaire or Survey Design	Role of the CRN	Primary Care
